# Pulmonary arterial hypertension caused by coadministration of dasatinib and voriconazole: A case report and literature review

**DOI:** 10.1097/MD.0000000000042927

**Published:** 2025-07-18

**Authors:** Chen Chen, Yan Dong, Ningxia Song, Jian Qi, Juandong Wang

**Affiliations:** a Department of Clinical Pharmacy, The Second Hospital of Shandong University, Jinan, China; b Department of Hematology, The Second Hospital of Shandong University, Jinan, China; c Department of Orthopedics, The 960th Hospital of PLA, Jinan, China; d Department of Breast Surgery, The Second Hospital of Shandong University, Jinan, China.

**Keywords:** case report, dasatinib, drug interaction, pulmonary arterial hypertension, voriconazole

## Abstract

**Rationale:**

It has been demonstrated that azole antifungal agents can significantly increase the plasma concentration of dasatinib. Therefore, it is recommended to reduce the dasatinib dose to 20 to 40 mg daily for patients receiving voriconazole. However, the safety of coadministering dasatinib and voriconazole remains unclear.

**Patient concerns:**

A 49-year-old woman with Philadelphia chromosome–positive acute lymphoblastic leukemia and invasive pulmonary aspergillosis reported dyspnea during therapy with reduced-dose dasatinib and standard-dose voriconazole.

**Diagnoses:**

Echocardiography revealed pleural effusion and a mean pulmonary arterial pressure of 40 mm Hg, suggesting pulmonary arterial hypertension.

**Interventions:**

Dasatinib was discontinued and flumatinib was initiated.

**Outcomes:**

The patient’s mean pulmonary arterial pressure was monitored and gradually normalized.

**Lessons:**

Despite recommendations to reduce the dasatinib dose to 20 to 40 mg daily for patients on voriconazole, the enhanced effect of dasatinib due to voriconazole coadministration may contribute to the development of pulmonary arterial hypertension. Regular echocardiography follow-up is essential for patients concurrently taking azole antifungal agents.

## 1. Introduction

Dasatinib is a second-generation tyrosine kinase inhibitor used for Philadelphia chromosome-positive chronic myeloid leukemia (Ph + CML) and Philadelphia chromosome-positive acute lymphoblastic leukemia (Ph + ALL). It targets BCR-ABL and SRC family kinases.^[1]^ Dasatinib is primarily metabolized by the cytochrome (CYP) CYP450 isoenzymes 3A4 in humans.^[2]^ Consequently, coadministration of CYP3A4 inhibitors or inducers may alter dasatinib plasma concentration through drug interactions.^[3]^ Azole antifungal agents, such as itraconazole, voriconazole, and posaconazole, which inhibit CYP3A4, can significantly increase dasatinib plasma levels.^[4]^ Although the prescribing information recommended that dose of dasatinib should be considered to decrease to 20 to 40 mg daily for patients taking administered with a strong CYP3A4 inhibitor, data on interactions with these inhibitors, particularly azole antifungals, remain limited. Given the widespread use of both dasatinib and voriconazole in patients with hematological malignancies, the safety implications of their coadministration warrant attention.

## 2. Case presentation

A 49-year-old female patient, with no history of cardiac disease or other disease, was diagnosed with Ph + ALL in May 2019. She underwent combination chemotherapy, including 400 mg of imatinib daily and hematopoietic stem cell transplantation. Imatinib was discontinued due to gastrointestinal reactions and dasatinib (100 mg daily) was started on January 14, 2021. After 7 months of dasatinib treatment, she developed invasive pulmonary aspergillosis and was given voriconazole, 6 mg/kg (400 mg) intravenously twice daily for 2 initial doses, followed by 4 mg/kg (200 mg) intravenously every 12 hours starting August 18, 2021, according to practice guidelines for the diagnosis and management of aspergillosis.^[5]^ Based on drug interactions between dasatnib and strong CYP3A4 inhibitors, her dasatinib dose was reduced to 20 mg daily. After 6 months of combined dasatinib and voriconazole treatment, she reported dyspnea. Echocardiography on February 22, 2022, showed pleural effusion and a mean pulmonary arterial pressure (mPAP) of 40 mm Hg. Dasatinib was discontinued and voriconazole was continued at 200 mg daily. Echocardiography showed a mPAP of 37 mm Hg on March 22, 2022. Dasatinib was restarted at 20 mg daily, but the patient increased the dose to 40 mg daily without medical advice. Echocardiography on April 26, 2022, showed an mPAP of 55 mm Hg. Dasatinib was discontinued again and voriconazole was continued at 200 mg daily until June 21, 2022. A repeat echocardiogram on July 17, 2022, showed a mPAP of 36 mm Hg. On December 22, 2022, echocardiography showed a mPAP of 22 mm Hg and flumatinib (600 mg daily) was started to treat primary disease.

**Figure 1.**
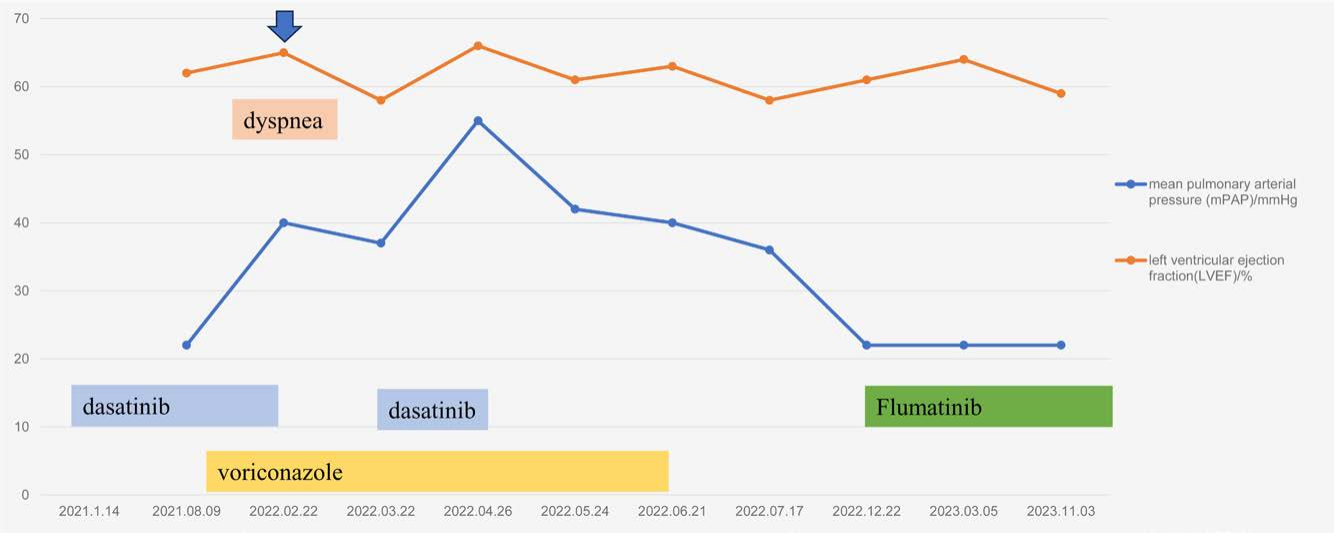
The timeline of the patient’s management and outcome.

During the 2-year follow-up period, the patient’s pulmonary artery pressure remained normal, and she experienced no dyspnea. The latest follow-up time on October 16, 2024, showed no discomfort. The patient’s management and outcomes are summarized in Figure 1. The detailed echocardiography indices are provided in Table 1.

**Table 1:** Findings at echocardiography.

Day	Mean pulmonary arterial pressure (mm Hg)	Left ventricular ejection fraction (%)
August 9, 2021	22	62
February 22, 2022	40	65
March 22, 2022	37	58
April 26, 2022	55	66
May 24, 2022	42	61
June 21, 2022	40	63
July 17, 2022	36	58
December 22, 2022	22	61
March 5, 2023	22	64
November 3, 2023	Not detected	59

## 3. Discussion

Dasatinib as a second-line tyrosine kinase inhibitor, inhibits BCR-ABL and other protein kinases more potently than imatinib. Cardiovascular events and pleural effusion occur more frequently with dasatinib compared to imatinib, and the incidence of pulmonary arterial hypertension in patients on long-term dasatinib treatment ranges from 0.2% to 5%.^[6–8]^ The exact mechanisms behind dasatinib-induced pulmonary arterial hypertension are not fully understood. One of these findings is that dasatinib-related pulmonary arterial hypertension is associated with Src protein kinase family which were crucial for TWIK (tandem of P domains in a weak inwardly rectifying K+ channel)-related acid sensitive potassium 1 potassium channel functioning.^[9]^ Decreasing TWIK-related acid sensitive potassium 1 activity caused by inhibition of SRC kinases resulted in intracellular calcium level increase thus enhancing vasoconstriction and vascular remodeling. Gover-Proaktor et al explored that dasatinib exposure caused disruption of endothelial barrier integrity and function, leading to impaired angiogenesis.^[10]^ Another reported mechanism of dasatinib’s effects involved direct toxicity to pulmonary artery endothelial cells through the production of mitochondrial reactive oxygen species.^[11]^ However, dasatinib-induced pulmonary arterial hypertension likely required a second risk factor.^[11]^ Several studies have supported to the “second hit” hypothesis that dasatinib may increase the risk of developing pulmonary arterial hypertension in the presence of an as-yet unrecognized second “hit.”^[12,13]^

We present a case of pulmonary arterial hypertension in a patient with Ph + ALL. The patient’s history and thorough workup did not suggest an alternative etiology. Pulmonary artery pressure remained normal during 7 months of dasatinib treatment at 100 mg daily. Despite the conflicting results from earlier studies on the dose-response relationship between dasatinib and vascular safety,^[8,12]^ our findings indicated that dasatinib at standard doses did not exhibit cardiotoxicity in this patient. Since dasatinib is primarily metabolized by CYP3A4, coadministration with moderate or strong CYP3A4 inhibitors increases dasatinib exposure.^[3,14]^ Given this drug interaction, the dasatinib dose for our patient was immediately reduced to 20 mg daily when voriconazole was also administered. After 6 months of coadministration of dasatinib (20 mg daily) and voriconazole, the patient initially reported dyspnea and developed pulmonary arterial hypertension, indicating that the enhanced effect of dasatinib due to the coadministration of voriconazole may have contributed to this adverse event. After discontinuing dasatinib, the pulmonary artery pressure decreased. During the treatment period, the patient failed to take dasatinib (40 mg/d) as prescribed by the doctor for 1 month, which directly led to the pulmonary artery pressure rising from 37 mm Hg before medication to 55 mm Hg after medication. These phenomena suggest that dasatinib remains the primary factor causing cardiovascular events, while voriconazole acts as a secondary contributor to the development of pulmonary hypertension.

There were few reports on the relationship between voriconazole and pulmonary toxicity or pulmonary arterial hypertension. However, Lee V showed that voriconazole increased oxidative stress in keratinocytes by directly inhibiting catalase leading to lower intracellular NADPH levels.^[15]^ Therefore, we hypothesize that the increased oxidative stress from voriconazole has potential clinical value as a “second hit” in causing dasatinib-induced pulmonary arterial hypertension. Pulmonary hypertension still occurred after standard 20 mg dasatinib dose reduction, which also indicates the existence of individualized differences, such as different pathological condition, interindividual variability of CYP450 isoenzymes and pharmacokinetic variability of voriconazole.^[8,16]^ Such individual differences complicate the prediction of dasatinib’s adverse reactions.

## 4. Conclusion

We present a case of a Ph + ALL patient who developed pulmonary arterial hypertension while on dasatinib and voriconazole, despite reducing the dasatinib dose to 20 to 40 mg daily. To our knowledge, this is one of the few cases of pulmonary arterial hypertension due to coadministration of dasatinib and voriconazole. Pulmonary artery pressure normalized after discontinuing both medications. Given the low incidence of dasatinib-related pulmonary arterial hypertension, echocardiography is not a routine screening method. Nevertheless, patients taking azole antifungal agents concurrently should have follow-up echocardiograms and optimized dasatinib dosing. Discontinuing dasatinib may reverse this adverse event if pulmonary arterial hypertension develops.

The article’s limitations include the speculative nature of voriconazole’s role as a “second hit” through oxidative stress, which is based on keratinocyte data. Future experiments are necessary for further verification. In addition, individual patient variability could also impact on this vascular safety issue observed in dasatinib-treated patients. Substantial work remains to be done to better understand the mechanisms by which dasatinib leads to pulmonary arterial hypertension.

## Author contributions

**Conceptualization:** Chen Chen, Ningxia Song, Juandong Wang.

**Data curation:** Yan Dong, Ningxia Song.

**Funding acquisition:** Chen Chen, Jian Qi.

**Methodology:** Ningxia Song, Jian Qi, Juandong Wang.

**Resources:** Ningxia Song.

**Software:** Yan Dong.

**Writing – original draft:** Chen Chen, Yan Dong, Ningxia Song, Jian Qi.

**Writing – review & editing:** Yan Dong, Ningxia Song, Jian Qi, Juandong Wang.
